# A gallotannin-rich fraction from *Caesalpinia spinosa *(Molina) Kuntze displays cytotoxic activity and raises sensitivity to doxorubicin in a leukemia cell line

**DOI:** 10.1186/1472-6882-12-38

**Published:** 2012-04-10

**Authors:** Diana M Castañeda, Luis Miguel Pombo, Claudia Patricia Urueña, John Fredy Hernandez, Susana Fiorentino

**Affiliations:** 1 Grupo de Inmunobiología y Biología Celular, Facultad de Ciencias, Pontificia Universidad Javeriana, Bogotá, Colombia, Carrera 7 N. 43-82 Building 52, Office 608; 2 Grupo de Farmacología Vegetal, Fundación Universitaria Juan N. Corpas, Bogotá, Colombia, Carrera 111 # 159A61

**Keywords:** Adjuvants, Gallotannins, *C.spinosa*, Tumor, Leukemia

## Abstract

**Background:**

Enhancement of tumor cell sensitivity may help facilitate a reduction in drug dosage using conventional chemotherapies. Consequently, it is worthwhile to search for adjuvants with the potential of increasing chemotherapeutic drug effectiveness and improving patient quality of life. Natural products are a very good source of such adjuvants.

**Methods:**

The biological activity of a fraction enriched in hydrolysable polyphenols (P2Et) obtained from *Caesalpinia spinosa *was evaluated using the hematopoietic cell line K562. This fraction was tested alone or in combination with the conventional chemotherapeutic drugs doxorubicin, vincristine, etoposide, camptothecin and taxol. The parameters evaluated were mitochondrial depolarization, caspase 3 activation, chromatin condensation and clonogenic activity.

**Results:**

We found that the P2Et fraction induced mitochondrial depolarization, activated caspase 3, induced chromatin condensation and decreased the clonogenic capacity of the K562 cell line. When the P2Et fraction was used in combination with chemotherapeutic drugs at sub-lethal concentrations, a fourfold reduction in doxorubicin inhibitory concentration 50 (IC_50_) was seen in the K562 cell line. This finding suggested that P2Et fraction activity is specific for the molecular target of doxorubicin.

**Conclusions:**

Our results suggest that a natural fraction extracted from *Caesalpinia spinosa *in combination with conventional chemotherapy in combination with natural products on leukemia cells may increase therapeutic effectiveness in relation to leukemia.

## Background

*Caesalpinia spinosa *is a shrub commonly named dividivi. It is acknowledged to have antimicrobial and antioxidant activity, and is traditionally known for its antitumor activity [1]. An ethanol extract from the fruit of *C.spinosa *has been proven to have antimicrobial activity against gram-positive and gram-negative bacteria, probably due to the presence of hydrolysable tannins in the fruits [2]. Hydrolysable tannins are a group of gallic acid esters associated with polyols (glucose, glucitol, shikimic acid, quinic acid and quercitol, among others), where the galloyl groups can be further cross-linked by etherification or oxidation to form complex structures. The gallotannins (gallic acid esters) are the simplest hydrolysable tannins, were 1,2,3,4,6-penta-O-galloyl-β-D-glucose (pentagalloyl glucose [PGG]) is the prototype and central compound of the biosynthetic pathway [3]. The presence of PGG, as well as gallotannins as mono, di or tri-galloylquinic acids, have been reported in *Caesalpinia *species corresponding to 40% to 60% of the fruit composition, depending upon their ecological habitat [4].

Gallic acid and its derivatives have proven selective antitumor activities, such as: reduction in biochemical markers associated with skin cancer [5]; cell death induction in several cancer cell lines, including leukemia [6,7], murine myeloma [8] and squamous carcinoma [9]. In addition, a beverage containing epigallocatechin gallate (EGCG) has been reported to promote tumor regression in patients with low-grade lymphomas [10]. Galloylquinic derivatives, such as 4,5-di-*O*-galloylquinic acid, have shown moderate cytotoxicity against melanoma cells (RPMI-7951) but not in other cell lines [11]. Extensive studies have been carried out on PGG and have demonstrated that this compound has a number biological activities related to cancer therapy and prevention, such as antiangiogenic, antiproliferative, anti-inflammatory and antioxidant [3]. However, there are limited studies supporting the use of polyphenols in the treatment of hematological malignancies, and even fewer involving hydrolysable polyphenols. In the present study, taking into account that polyphenol antioxidant activity has been clearly implicated in the control of these malignancies [12], and that *C.spinosa *has a high content of polyphenols and is widely distributed in our country, we have evaluated the anti-tumor activity of *C.spinosa *pod extracts and complex fractions using the erythroleukemia cell line (K652) as a model of hematological malignancy.

## Methods

### Plant material

*C.spinosa *pods were collected in Villa de Leyva, Boyacá, Colombia in March 2007 and identified by Luis Carlos Jiménez from the Colombian National Herbarium; voucher specimen number COL 523714.

### Plant extraction and purification

Three kg of fresh pods from *C.spinosa *were dried under airflow in a solar oven at 35°C and ground down to obtain 1.8 kg of plant material. Subsequently the plant material was extracted with ethanol (96%, 10 L) in a recirculating percolator (twice per day) over a period of 10 days. The ethanol crude extract (80 g) was concentrated under vacuum, trapped on silica gel and excess humidity removed at 25°C. Afterwards, the ethanol extract was fractionated with the following solvents: petroleum ether (1.5 L); chloroform (2 L); ethyl acetate (2 L); ethanol (2 L) and water (2 L) (aqueous fraction). From the ethyl acetate fraction we obtained an abundant precipitate which we named (P2Et). This corresponded to 2.78% of the ethanol extract and a supernatant which we named (S2Et) corresponded to 1.11%. The P2Et, S2Et and aqueous fractions were selected for biological testing based on their cytotoxic activity. The extraction protocol was performed three times and the chromatographic profiles of the components were verified.

The quality control carried out on the P2Et fraction gave the following results: foreign matter less than 2%; total ash less than 8%; ash that was insoluble in hydrochloric acid less than 1%; no evidence of heavy metals and pesticides. These result met the British Herbal Pharmacopoeia quality parameters.

### Phytochemical characterization

Fraction characterization was determined by means of standard phytochemical tests. In the total ethanol extract the presence of alkaloids or nitrogen compounds were not identified using Dragenddorff, Valser, Reineckate and Mayer's reagents. The Shinoda test (Mg in HCl) was positive suggesting the presence of flavanones, flavanonols, flavones, flavonols or isoflavones. Hydrogen peroxide evidenced the presence of naphthoquinones and/or anthraquinones. The presence of steroids was demonstrated using Liebermann Burchard reagent. Low concentrations of steroidal saponins and/or triterpenoids were detected using hemolysis and foam tests. In order to assess the presence of anthraquinone glycosides, Borntrager's reaction (treatment with ammonia solution) was used. The presence of tannins was verified using ferric chloride solution, gelatin and lead acetate [13]. P2Et, S2Et and aqueous fractions exhibited the presence of leucoanthocyanidins, the absence of quinones and a significant tannin content, especially in P2Et fraction.

### Thin layer chromatography (TLC)

Chromatographic analysis was carried out on TLC aluminum sheets (10 × 5 cm) (Merck) silica gel 60 F 254. Three solvent systems were used: Petroleum ether - ethyl acetate - formic acid (40:60:1); chloroform - ethyl acetate - acetic acid (50:50:1); and toluene - acetonitrile - formic acid (70:30:1). After basic hydrolysis, the P2Et and S2Et fractions were dissolved in methanol (1%) and detected using UV (254 nm), FeCl_3 _(10%) and vanillin-sulfuric acid (VS)/110°C. Gallic acid was used as a positive control.

### HPLC - PDA-MS

HPLC analysis was carried out in an Alliance 2795 (Waters^®^, UK) with a PDA detector (996). A Sunfire (Waters) column C18 - 2.1 × 150 mm × 5 μm was used, with a flow rate of 0.25 ml/min and a linear gradient from 95% solvent A (H_2_O + 1%CH_3_COOH) and 5% solvent B (CH_3_CN) to 60% in solvent A and 40% in solvent B, over a period of 25 min. The mass spectrum (MS) analysis was carried out using a LCT (Micromass^®^, UK) mass spectrometer with an ESI source. The percentage relative abundance was determined using quercetin as an internal standard (0.0625 μg/μl). Runs were performed in triplicate.

### Tumor cell line and normal cells

The cell lines used as cancer cells were K562, a human erythroleukemia and MCF7, a human breast adenocarcinoma, from the American Type Culture Collection (ATCC); and the cell lines used as normal cells were human peripheral blood mononuclear cells (PBMC) and human fibroblasts obtained from normal healthy donors after informed consent was given. This project was approved by the ethics committee (founded in 2002) of the Science Faculty at a meeting on August 21, 2007. The culture conditions under which the cell lines were maintained have already been reported [14].

### *In vitro *cytotoxicity assays

The cytotoxic effects of the fractions and conventional chemotherapeutic drugs (doxorubicin, etoposide, vincristine and taxol) were evaluated using normal and tumor cells by means of trypan blue and the methylthiazol tetrazolium (MTT) assay, as previously reported [14]. The P2Et fraction was dissolve in ethanol and the corresponding vehicle was used as a negative control.

### Measurement of mitochondrial membrane potential

The cells were treated with different concentrations of the P2Et fraction or valinomycin (positive control, 0.1 μg/ml) for 4, 8 and 12 h for K562 cells, and for 6, 12 and 24 h for MCF7 cells. The mitochondrial membrane potential (MMP) was measured using JC-1 dye, as previously described [14].

### Annexin V assay

Phosphatidylserine (PS) externalization was assessed by flow cytometry using Annexin V-FITC (Molecular Probes, Invitrogen Corp, Carlsbad, CA, USA)/PI (Sigma, Saint Louis, MO, USA). K562 and MCF7 cells (3 × 10^5^) were treated with doxorubicin, ethanol or the P2Et fraction for 48 h. After treatment, cells were suspended in Annexin buffer (Hepes 100 mM, NaCl 140 mM, CaCl_2 _2.5 mM) and incubated with Annexin V-FITC for 8 min at room temperature. Then the cells were incubated with PI for 2 min at 4°C, acquired on a FACSAria I (Becton Dickinson, New Jersey, USA) and analyzed with FlowJo software (Tree Star Inc., Ashland, USA). Results are expressed as the mean ± SE of three independent experiments.

### Caspase 3 assays

Caspase 3 activity was estimated using the caspase 3 colorimetric assay kit, which detects enzyme activity based on the cleavage of Asp-Glu-Val-Asp (DEVD)-pNA (R&D Systems Inc., Minneapolis, MN, USA). Briefly, cells (2 × 10^5 ^cells/ml) were cultured using different concentrations of the P2Et fraction and doxorubicin (positive control) or ethanol (negative control) for 48 h. After the cells were ice lysed for 10 min the enzyme activity was measure on 96-well flat-bottom microplates with 50 μl of supernatant. The supernatant was prepared by centrifuging at 10,000 × g for 1 min (100-200 μg of total protein), and then adding 50 μl of reaction buffer supplemented with 10 μl of DTT and 5 μl of caspase 3 colorimetric substrate DEVD-pNA. Next cells were incubated for 1 ± 2 h at 37°C and caspase-3 activity was measured at 405 nm on a spectrophotometer (Multiskan Labsystem). The increase in caspase 3 activity was calculated relative to the absorbance value of the negative control.

### DNA fragmentation and cell cycle analysis

DAPI (4',6-diamidino-2-phenylindole, Sigma) stained cells were monitored under a microscope as previously described [14]. Slides were mounted using prolong anti-fade kit (Molecular Probes, Eugene, Oregon, USA) and cells were analyzed under a fluorescence microscope (Olympus, Japan). Cell cycle analysis was undertaken as previously reported [14].

### Clonogenic assays

The clonogenic assays were performed as previously described [14]. Briefly, K562 human cells (2.5 × 10^5 ^cells/well) were plated (96-well plate) and treated with the P2Et fraction at 40 and 20 μg/ml, or with 15 and 6 μg/ml etoposide, or 0.2% ethanol (in PBS) and incubated for 24 h under a humidified environment at 37°C and 5% CO_2_. After treatment cells were re-plated onto 0.5% agar dishes (60 mm, 20,000 cells/dish), incubated for 14 days (37°C and 5% CO_2_) and stained with violet crystal (0.4% in ethanol). Cell colonies with more than 50 cells were counted. Treatments were performed in triplicate, and results expressed as mean ± SE.

### P2Et fraction adjuvant activity

P2Et fraction adjuvant activity was assessed using K562 and MCF7 cells in combination with the well-known cancer treatment drugs doxorubicin, vincristine, taxol and camptothecin. Cell viability was evaluated by means of the MTT assay. K562 and MCF7 cells (5 × 10^3 ^cells/well) were seeded in 96-well plates and treated for 6 h with sublethal concentrations of the P2Et fraction (1.6 μg/ml, 27 fold less than the IC_50 _value for K562 cells and 15.5 μg/ml for MCF7 cells); washed and incubated in fresh medium with each drug for 48 h at 37°C in humid atmosphere and 5% CO_2_. Sublethal concentrations of the chemotherapeutic drugs had been previously determined by MTT assay. Results are expressed as cell viability percentage relative to the control (100 × Treatment OD/Negative control OD).

### Statistical analysis

Data is presented as the mean ± SE. The data were analyzed by one- and two-way ANOVA and differences between control and treated groups were determined using the Bonferroni and Tukey tests. Differences were considered significant for p < 0.05 and were determined using the GraphPad prism 5.0 software.

## Results and discussion

### TLC and HPLC-PDA-MS analyses

**Figure 1:**
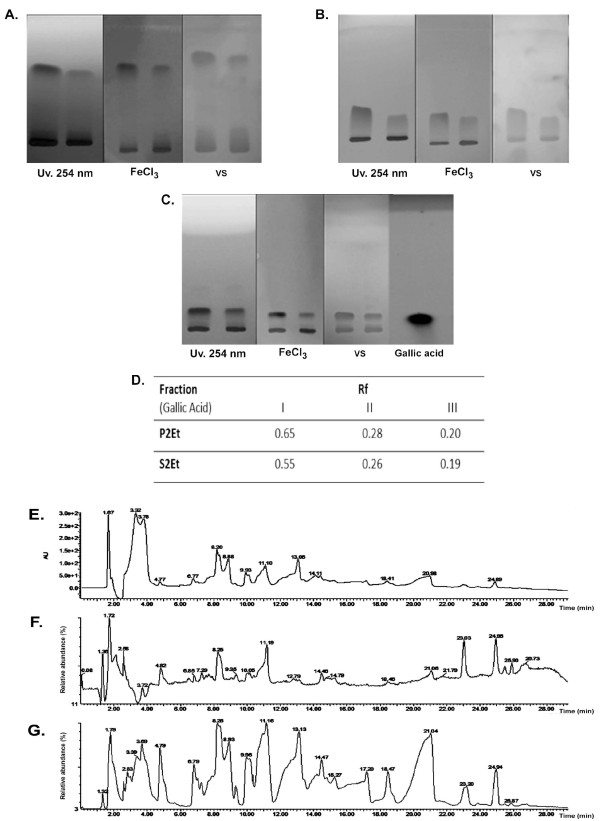
**P2Et fraction thin layer chromatographic profile**. (A) Solvent I: petroleum benzene - ethyl acetate - formic acid (40:60:1). (B) Solvent II: chloroform - ethyl acetate - acetic acid (50:50:1). (C) Solvent III: Toluene - acetonitrile - formic acid (70:30:1). Lane 1: P2Et fraction basic hydrolysis products. Lane 2: S2Et fraction basic hydrolysis products. TLC visualization with UV, 254 nm, ferric chloride (FeCl_3_) and vanillin/sulfuric acid (VS) sprays. Gallic acid is a positive control. (D) Gallic acid Rf values in different solvent systems. (E) HPLC chromatogram at 263 nm. (F) MS spectrum positive mode. (G) MS spectrum negative mode.

TLC analysis of the P2Et and S2Et fractions after hydrolysis revealed the presence of gallic acid visualized using UV (254 nm), ferric chloride and vainillin-sulfuric acid (VS) reagents (Figure 1A, B and 1C). Gallic acid retention (Rf) values in different solvent systems are shown in Figure 1D. The values are in agreement with those previously reported [15].

Chemical characterization of the most biological active fraction, P2Et, was carried out. The presence of gallic acid (after hydrolysis) was confirmed (data not shown) using proton nuclear magnetic resonance (H-NMR) spectroscopy. HPLC-PDA analysis revealed 17 peaks at 263 nm, and peaks having the highest absorbance display retention times of 3.32 and 3.78 min (Figure 1E). The peak molecular weights were calculated by means of mass spectrometry, and seven compounds were found to match with the molecular weights of compounds already published [4] for this species (Figure 1F and 1G, and Table 1). Clifford et al. reported the presence of gallotannins, ellagitannins, and galloylquinic acid tannins in *C.spinosa *fruits [4]. In summary, the P2Et fraction was found to contain hydrolysable tannins such as galloylquinic acid derivatives at high percentages, and pentagalloylglucose and gallic acid-like compounds (gallates) at lower percentages.

**Table 1:** Compounds in the P2Et fraction.

MS (m/z)	Reported m/z	Possible compounds	Relative abundance* (%)
**344**	344	1-O-galloylquinic acid	162,76
	344	3-O-galloylquinic acid	
	344	4-O-galloylquinic acid	
	344	5-O-galloylquinic acid	
**496**	496	3,4-di-O-galloylquinic acid	38,13
	496	3,5-di-O-galloylquinic acid	
	496	4,5-di-O-galloylquinic acid	
	648	1,3,4-tri-O-galloylquinic acid	
**648**	648	1,3,5-tri-O-galloylquinic acid	15,73
	648	1,4,5-tri-O-galloylquinic acid	
	648	3,4,5-tri-O-galloylquinic acid	
**991**	991	Pentagalloylglucose	7,43
**322**	322	Digallate	15,82
**183**	183	Methyl gallate	15,98
**335**	335	Methyl digallate	3,14

M/z ratio of *C.spinosa *reported compounds and compounds from the P2Et fraction matching the m/z ratio. The relative abundance is expressed as % of quercetin (internal standard)

### Morphological and cell viability changes in the K562 tumor cell line induced by fractions derived from *Caesalpinia spinosa*

**Figure 2:**
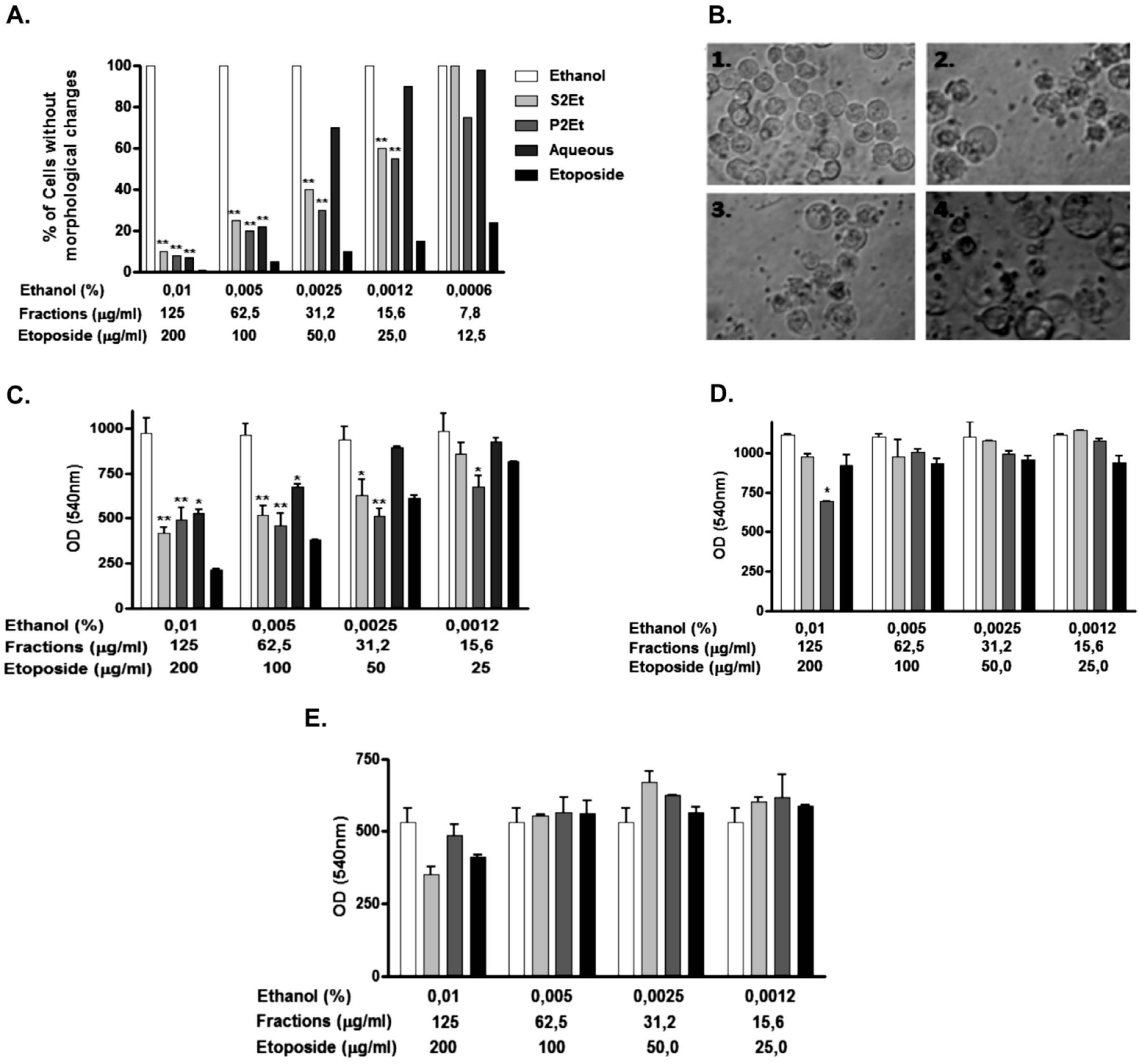
**Induction of morphological changes and cytotoxicity in K562 cells by C. *spinosa *fractions without affecting normal human cells**. K562 (A, B, C), Fibroblasts (D), or PBMCs (E) cells were treated with *C.spinosa *fractions at different concentrations for 48 h. Morphological changes were evaluated by direct observation using a light microscope at a magnification of 40X (B: 1. Ethanol; 2. S2Et; 3. P2Et; and 4. Etoposide). Cell cytotoxicity was estimated using the MTT assay (C, D, and E). Ethanol and etoposide were used as negative and positive controls, respectively. The results of six experiments are shown.

Assessment of changes in K562 cell viability was carried out using trypan blue to select fractions derived from *C. spinosa *that had potential antitumor activity. Antitumor activity was found using the aqueous fraction at a concentration of 62.5 μg/ml, while the S2Et and P2Et fractions showed antitumor activity at a concentration of 15.6 μg/ml (Figure 2A). Assessment of changes in the appearance of intracellular vesicles, cell membrane morphology, nuclear alterations and the presence of necrotic cells were carried out by means of direct observation using a light microscope, and compared with etoposide activity as shown in Figure 2B.

In order to confirm cell viability the activity of mitochondria dehydrogenase was estimated using the MTT assay. The aqueous fraction induced cytotoxicity at a concentration of 62.5 μg/ml, while the S2Et and P2Et fractions induced cytotoxicity at concentrations of 31.2 and 15.6 μg/ml, respectively (Figure 2C). The P2Et fraction displayed significant cytotoxic activity in a dose-dependent manner. This finding was in agreement with those reported in previous studies on leukemia cells using chemically related P2Et tannins. Alkyl gallates and gallamides have been shown to exhibit cytotoxic activity with regard to murine lymphocyte leukemia cells (L1210) [6], and an acetone derived gallotannin-enriched fraction extracted from *Eugenia jambos *species was found to have a cytotoxic effect on promyelocytic leukemia HL-60 cells [16]. The biological activity of tannins has been widely documented [6,17], but reports related to *C. spinosa *anti-tumoral activity are scarce. Gali-Muhtasib et al. found that hydrolysable tannins extracted from the fruit of *C. spinosa *decreased the level of skin cancer biochemical markers. In addition, there are reports describing anti-inflammatory and anti-microbial activity of tannins [5].

Additionally, the cytotoxic activity of *C. spinosa *fractions was evaluated using gingival fibroblasts from the oral mucosa or normal human mononuclear cells. The S2Et and aqueous fractions did not show cytotoxic activity with regard to the PHA-stimulated PBMCs or fibroblast cells, and the P2Et fraction had a slight cytotoxic effect at a concentration of 125 μg/ml on fibroblasts (Figure 2D and 2E). The IC_50 _values are shown in Table 2. However, it has been reported that 1-*O*-galloyl castalagin, casuarinin and gallotannins extracted from *Eugenia jambos *are cytotoxic to lymphocytes and to normal hepatic cells [16,18]. The decreased toxicity to normal cells and the specificity toward tumor cells was probably due to interactions among the fraction compounds. Evaluation of biological activity was continued only in the case of the P2Et fraction due to its superior biological results and the remarkable yield obtained during the extraction procedures.

**Table 2:** IC_50 _values of *C.spinosa *fractions and drugs in tumor cell lines and normal cells (gingival fibroblasts and PBMC) estimated using the MTT assay. *Concentration (μg/ml) ± SE

Cell line/Treatment	K562	Fibroblast	PBMC
S2Et	> 125*	> 125*	> 125*
P2Et	44.50* ± 4.05	64.30* ± 1.00	> 125*
Aqueous	> 125*	> 125*	> 125*
Etoposide	15.60* ± 0.31	> 200*	> 200*
Doxorubicin	0.24* ± 0.14	5.80* ± 0.40	2023* ± 0.50
Taxol	1.14* ± 0.15	-	-
Vincristine	1.07* ± 0.28	0.07* ± 2.40	0.18* ± 2.00
Camptothecin	14.10* ± 3.70	-	-

### Induction of MMP loss, caspase 3 activation and DNA fragmentation in the K562 cell line by the P2Et fraction

**Figure 3:**
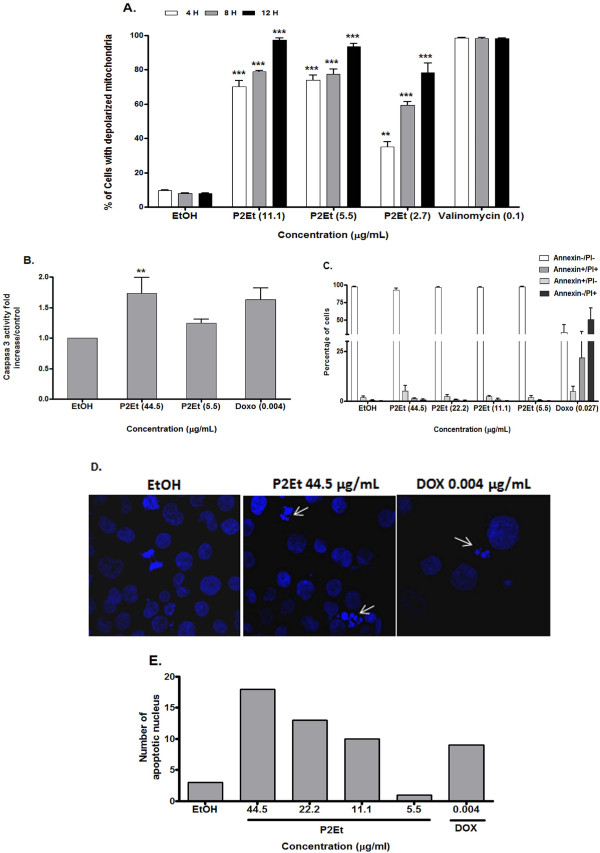
**P2Et fraction induction of MMP loss, activation of caspase 3 and DNA fragmentation**. (A) K562 cells treated with the P2Et fraction for 4, 8 or 12 h, stained with JC-1 and analyzed by flow cytometry. Ethanol was used as negative and valinomycin as positive controls (*** p < 0.001). (B) Caspase 3 activity was measured using the enzyme-linked immunosorbent assay (ELISA) after cell treatment with the P2Et fraction or doxorubicin (positive control) for 48 h (** p < 0.005). (C) PS externalization was estimated in K562 cells using flow cytometry after cell treatment for 48 h with the P2Et fraction, doxorubicin (positive control) or ethanol (negative control). (D) DNA was stained with 4',6-diamidino-2-phenylindole (DAPI) after cell treatment for 48 h. Apoptotic cells or fragmented nuclei were observed under fluorescent microscope (40X). (E) Apoptotic cells were estimated by random observation in six fields and by counting cells with strong fluorescence or fragmented nuclei.

Cell death analysis may help envisage the possible uses of the P2Et fraction in cancer treatment. Cytotoxic drugs can induce apoptosis by means of the mitochondrial pathway, inferring with MMP loss, effector caspases activation and DNA fragmentation [19]. In order to determine if the P2Et fraction induces apoptosis and changes in MMP, K562 cells were treated with the P2Et fraction for 4, 8 and 12 h. Results showed that MMP loss occurred in a dose-dependent manner, starting at 4 h and continuing over a 12 h period (Figure 3A). Consequently, effector caspases activation may occur, which in fact was demonstrated in K562 cells after 48 h of treatment (Figure 3B). Finally, only a low percentage of cells displayed PS externalization (Figure 3C), suggesting that the loss in MMP was not followed by PS externalization; this was probably because the cells entered a different cell death process. Nonetheless, an early loss of MMP seems to be followed by DNA fragmentation (Figure 3D and 3E).

Loss of MMP induced by the P2Et fraction can be seen as an early apoptotic event that lasts for 12 h. These results correlated with previously described activity for alkyl gallates and gallamides having 8 to 12 carbon chains [6,18]. In addition, the P2Et fraction induced caspase 3 activation and DNA fragmentation, suggesting that after MMP loss, apoptotic cell death may be ongoing. However, since evidence of PS externalization was not found (Figure 3D), the decrease in long-term viability might be mediated by other cell death mechanisms.

### Effect of the P2Et fraction on tumor cell clonogenic capacity

**Figure 4:**
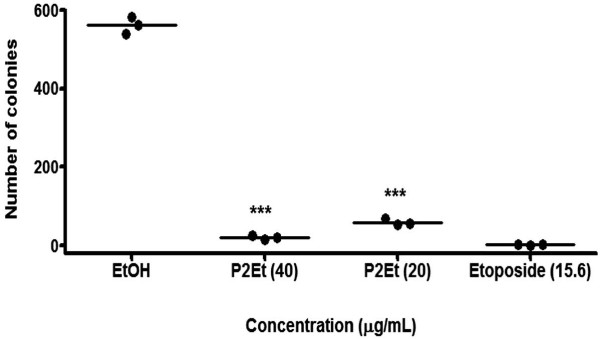
**Abrogation of clonogenic activity in K562 cells by the P2Et fraction**. Cells were treated with the P2Et fraction, ethanol as negative control and etoposide as positive control. Data represents the mean number of colonies ± SE of 3 independent experiments. ***(p < 0.001) versus control.

Clonogenic ability is considered to be a gold standard test for determining the antitumor activity of extracted plant fractions as well as isolated compounds [20]. K562 cells were treated with the P2Et fraction for 6 h and agar cultured for 14 days. Experimental data showed a significant decrease in the clonogenic ability of treated tumor cells as compared with the negative control (Figure 4). Indeed, a decrease in clonogenic ability also implies a decrease in long-term viability. Similar results were reported for PGG, a compound also present in the P2Et fraction, which is able to induce caspase-mediated apoptosis in the HL-60 cell line [21].

### Adjuvant activity of the P2Et fraction

**Figure 5:**
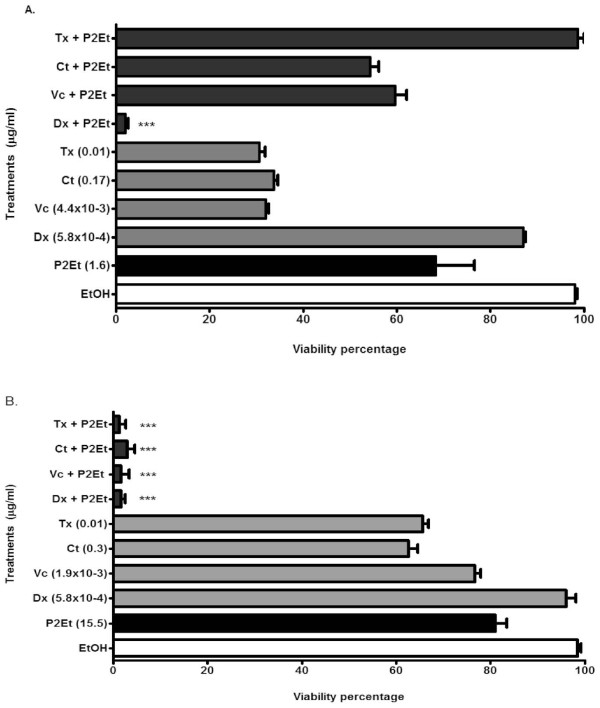
**Adjuvant activity of the P2Et fraction on the K562 and MCF7 cell lines**. K562 (A) or MCF7 (B) cells were treated with the P2Et fraction or ethanol (negative control) for 6 h, washed and treated with doxorubicin (Dx), vincristine (Vt), camptothecin (Ct) or taxol (Tx) at the concentrations shown over a 48 h period. Cell cytotoxicity was estimated using the MTT assay. Data represent three independent experiments. *** (p < 0.001).

**Figure 6:**
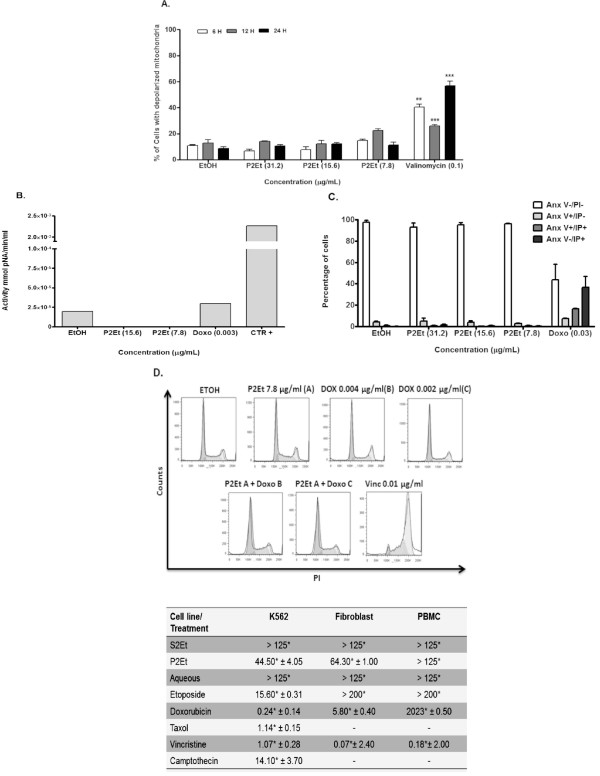
**Induction of G1 cell cycle arrest but not apoptotic cell death in MCF7 cells by the P2Et fraction**. (A) MCF7 cells treated with the P2Et fraction for 6, 12 or 24 h, stained with JC-1 and analyzed by flow cytometry. Ethanol was used as negative and valinomycin as positive controls (*** p < 0.001). (B) Caspase 3 activity was measured using the ELISA assay after cells had been treated with the P2Et fraction or doxorubicin (positive control) for 48 h (** p < 0.005). (C) PS externalization was estimated in MCF7 cells using flow cytometry after treatment with the P2Et fraction, doxorubicin (positive control) or ethanol (negative control) for 48 h. (D) Cell cycle analysis was performed on MCF7 cells treated with ethanol (negative control), the P2Et fraction (7.80 μg/ml (A)), doxorubicin (0.004 μg/ml (B) or 0.002 μg/ml (C)), the P2Et fraction (A) + doxorubicin (B or C) and vincristine (0.01 μg/ml, positive control) for 24 h. The cells were permeabilized, stained with propidium iodide (PI), acquired on a FACSAria I and analyzed with FLowJo software. Histograms represent relative cell DNA content measured in three independent experiments.

In order to determine if the P2Et fraction improves tumor cell sensitivity to the conventional cancer drugs doxorubicin, vincristine, camptothecin and taxol, a dose-response test was used to choose sub-optimal drugs and fraction concentrations as described in the Methods section. Separately, doxorubicin or the P2Et fraction at the tested concentrations showed a slight effect on cell viability, but when used in combination a significant decrease in cell viability was observed. In contrast, when treatment with the P2Et fraction was combined with vincristine or camptothecin, no positive effect was identified (Figure 5A). Therefore, it is worthwhile to point out that pretreatment with the P2Et fraction may be enhancing the cytotoxicity of taxol, highlighting a possible deleterious activity on taxanes when combined with the polyphenol fraction. On the contrary, in a similar analysis carried out on the breast cancer cell line MCF7, the P2Et fraction did exhibit adjuvant activity when combine with all the tested drugs (doxorubicin, vincristine, taxol and camptothecin, Figure 5B). Apoptotic cell death may not be associated with treatment with the P2Et fraction, since MMP loss and caspase 3 activity were not detected (Figure 6A and 6B) nor PS externalization found (Figure 6C). Instead, a slight cell cycle arrest in the G1 phase was observed when the P2Et fraction and doxorubicin treatments were combined at two different concentrations (Figure 6D), unlike the results achieved with the K562 cell line. The latter results suggest that P2Et adjuvant activity depends on the type of anticancer drug and the cancer cell line.

The use of natural products as adjuvants in antitumor therapy has been previously addressed; indeed, EGCG, a condensate tannin present in green tea, has been shown to sensitize cancer cells to taxol treatment in a breast cancer model [22]. Also, it has been reported that EGCG (at low doses) and epicatechin gallate are not cytotoxic to a chemoresistant hepatocellular carcinoma, but in combination with doxorubicin, tumor cells were sensitized and a decrease in tumor size was seen *in vivo *using a doxorubicin-resistant liver cancer model [23]. The *C. spinosa *fraction that was enriched in gallotannins not only exhibited cytotoxic activity, but also activity associated with conventional anti-cancer drugs, demonstrating once again the efficiency of natural products as potential sources of adjuvants that can be used in antitumor therapy. Herein, we described a *C.spinosa *derived fraction, P2Et, that was relatively easy to produce and which offered high yields in the extraction procedures; and preliminary cytotoxicity studies suggest that it can be used at very low dosages. In studies using several other cell lines (data not shown) a wide range of activities may be foreseen. Currently, the *in vivo *antitumor activity of the P2Et fraction is being evaluated and chemical characterization is being completed so that the molecular interactions may be scrutinized; and so that the cell death mechanism and the sensitization exerted by this fraction can be elucidated.

## Conclusions

Our results suggest that the therapeutic efficacy of conventional chemotherapeutic drugs when combined with a blend of natural polyphenols may be increased in relation to leukemia or breast cancer cells. However, the molecular mechanisms underlying this activity have yet to be explained and are currently under study at our laboratory.

## Competing interests

The authors declare that they have no competing interests.

## Authors' contributions

The present work was conceived, directed and coordinated by SF. *In vitro *cytotoxicity assays, MMP measurements, Annexin V determinations, clonogenic assays, cell cycle analysis and cell line maintenance was undertaken by DC. Caspase 3 activity analysis and the DAPI DNA fragmentation test was carried out by CU. Plant fraction preparation, characterizations and statistical analysis were conducted by LMP. JH collaborated in the writing of the manuscript and analysis of the results. All of the authors have read the manuscript and agree with its contents.

## Pre-publication history

The pre-publication history for this paper can be accessed here:

http://www.biomedcentral.com/1472-6882/12/38/prepub
